# Wavelet-based visual compass

**DOI:** 10.1371/journal.pone.0344575

**Published:** 2026-04-07

**Authors:** Stefan Meyer, James C. Knight, Alexander Dewar, Efstathios Kagioulis, Thomas Nowotny, Paul R. Graham, Andrew Philippides

**Affiliations:** 1 Sussex AI, School of Engineering and Informatics, University of Sussex, Brighton, United Kingdom; 2 Information & Communication Technologies, Imperial College London, London, United Kingdom; 3 Sussex Neuroscience, School of Life Sciences, University of Sussex, Brighton, United Kingdom; Chunghwa Telecom Co. Ltd., TAIWAN

## Abstract

For many ant species, successful visual navigation is crucial for the survival of the individual and the colony, meaning these small-brained insects have evolved to be exceptional navigators. This makes them an ideal inspiration for biomimetic robotics research. Visual compass-style snapshot models have been used to model visual navigation in ants and have been applied to visual teach-and-repeat style robot navigation. In these models, images or ‘snapshots’ stored when the ant first travels a route are compared to views experienced when recapitulating the route to derive a bearing that will direct the ant along the route (rather than navigating to a discrete goal location as in visual homing). While the majority of visual-compass snapshot models have used raw images, we have shown in preliminary work that visual pre-processing by Haar wavelets that quantify spatial frequencies at every location in an image can improve the snapshot robustness. These wavelets effectively filter images for oriented edges at certain spatial frequencies in a way that mimics the processing seen in natural visual systems. Here, we extend our findings by investigating the properties and limits of bearing recovery in the face of naturalistic perturbations, focusing on comparing wavelets with edge-processed or raw images of different resolutions. We find that: (1) high frequency localised wavelet coefficients highlight distant objects; (2) The effect disappears when the resolution is decreased, as far away objects blur together; (3) If navigating using visual-compass style snapshot navigation, perturbations in the environment can be compensated solely by choosing suitable image processing. Our work extends the corpus of research on spatial frequency-based encodings for snapshot navigation, which has mainly focused on non-localised encodings (such as Fourier Transforms) applied to visual homing. We do this by providing an in-depth analysis of localised spatial-frequency encodings and their dis-/advantages for route following via visual compass style bearing recovery.

## Introduction

Navigation is a crucial task for many animal species, though some animals are more proficient navigators than others. Central place foragers that venture out to forage and return to a fixed location (e.g., a nest) are particular experts in navigation. If a food source is profitable and long-lasting, then an individual will make multiple traversals of the route connecting the nest and food locations, a behaviour known as route fidelity, of which desert ant foragers are a well-known example [1,2]. Because route traversal is essentially the sole job of these foragers, and because successful foraging is critical to the success of the colony, evolution has equipped these ants with robust navigation strategies efficient enough for animals with brains of only a million neurons [3]. The way that ants navigate has therefore inspired biomimetic researchers seeking efficient methods for navigating robots. In particular, several models have been developed for navigating one-way routes that use views for bearing recovery, which are inspired by ant route-following behaviour and the neural structures known to be involved in navigation [4–10]. As ant foragers’ eyes have also been optimised by evolution, here we ask whether bio-inspired visual processing based on wavelets can aid bearing recovery in the face of naturalistic perturbations. Because bearing recovery is the base behaviour driving familiarity-based route navigation algorithms, this will tell us whether biomimetic visual processing is useful for robots navigating routes through natural environments. Further, by assessing the contributions of oriented edges at different spatial scales to bearing recovery, we can start to understand what features of the visual world are useful to navigating robots and perhaps the ants that inspire them.

Experimental evidence suggests that insect eyes implement a system of simple visual filters [11,12], which process views to compressed representations formed by the filter outputs. These filters can be thought of as some form of oriented edge-detection but crucially at different spatial scales meaning that, for visual navigation, they have been emulated by spatial-frequency based approaches including Fourier [13], Zernike [14] and Wavelet [15] transforms. We use the wavelet transform because, unlike for instance a standard Fourier representation which provides information on how much of each spatial frequency occurs in an image, wavelets allow one to localise the frequencies within the image, so that the result of the transform can be viewed and used as an image. Further, in the sparse two-dimensional version we use, the transform naturally extracts vertical, horizontal and diagonal elements by convolving the input signal with discretely shifted and scaled wavelet functions [15]. We chose filters corresponding to ‘Haar’ wavelets [16] which are plausible approximations to filters implemented in an insect’s visual system as they respond strongly to edges and are well localised in space. In general terms, this means that each filter response yields a coefficient of magnitude representing how well edges at each location in the image match the orientation and scale of the Haar filter.

As mentioned, wavelets are only one example of spatial-frequency-based encoding and others have been used for snapshot-based visual homing. Fourier encoded views have been used in robotics in the context of place recognition in the past (e.g., [17]) and adapted for visual homing [18,19]. The model of [18] has also been used recently on a small robot where the visual homing element is used to correct odometric based navigation [20]. Extending these findings, Zernike moments have been found to change between locations in a way that allows the derivation of a homing direction [21,22] and similar results have been reported for homing based on a subset of Haar wavelet responses [23].

However, these works focus on visual homing, that is, getting to a single goal location from a catchment area around that goal. In contrast, we are interested in the utility of frequency based visual processing for bearing recovery when using a snapshot as a visual compass, because this is the way that ants navigate routes. Both homing and route navigation are necessary (for ants and robots), but the visual information is used in different ways and can lead to different results. For instance, Stürzl and Zeil [24] showed that while processing images into edges (via difference of Gaussians) was useful for visual homing with snapshots it destroyed much of the useful information for visual compass models. Crucially, the results for a visual compass method depends on what resolution is used as the final output image, as Kagioulis et al. [25] showed that a low-resolution edge-processed image could be more robust than raw images for visual route following. The interplay between resolution and edges is particularly pertinent as much of the work on goal searching with snapshot-based methods using frequency-encodings showed that low spatial frequency representations are especially useful [21,22,26]. However, our previous work [27] (albeit based on a simplified simulation of an ant habitat) does not confirm the finding of low-frequency superiority for recovering a bearing as part of visual route navigation.

In this paper, we therefore investigate how the details of localized-frequency encoding affect the robustness of a visual compass in a realistic 3D reconstruction of a natural ant habitat. We compare across resolutions between 0.5 and 4 degrees/pixel that could be plausibly achieved after filtering by insect eyes [28]. In previous work, we saw that for images with no visual filtering, route navigation was optimal for resolutions around 1–2° [29]. Here we focus instead on edge information obtained by two different processes – Canny edge detection and wavelet decomposition – and compare results to a pixel-based encoding. Following and extending [27], we look at the advantages and disadvantages of frequency-based encoding for navigational performance under different environmental circumstances and perturbations, such as those caused by ground tilt and visual obstruction.

## Methods

In this work, we investigate how localized frequency information from panoramic images, or snapshots, can be used to recover the correct bearing in the vicinity of a snapshot location. We use a simulated world with conditions representing uneven ground and occluding objects. We evaluate the quality of a visual representation by assessing the distance from the snapshot location at which the bearing can be reliably recovered (catchment area). In what follows we will first describe the world and how we manipulate it, then how views can be used as a visual compass to derive a bearing and finally the different image representations we investigated.

### Determining useful information for navigation using a visual compass

When using a visual compass, a navigating agent in the vicinity of a reference location where a snapshot was taken rotates on the spot and compares the current view with the snapshot, thus sampling the rotational image difference function (RIDF). The direction the agent faces when the match between view and snapshot is best – or the RIDF is at its global minimum – is an estimate for the correct bearing. The success of this method is measured by the angular error between estimated and original bearing. This error typically increases with the distance between the current location and the reference location. A robust visual compass is characterised by a large maximum distance from which an accurate bearing can be recovered.

**Fig 1:**
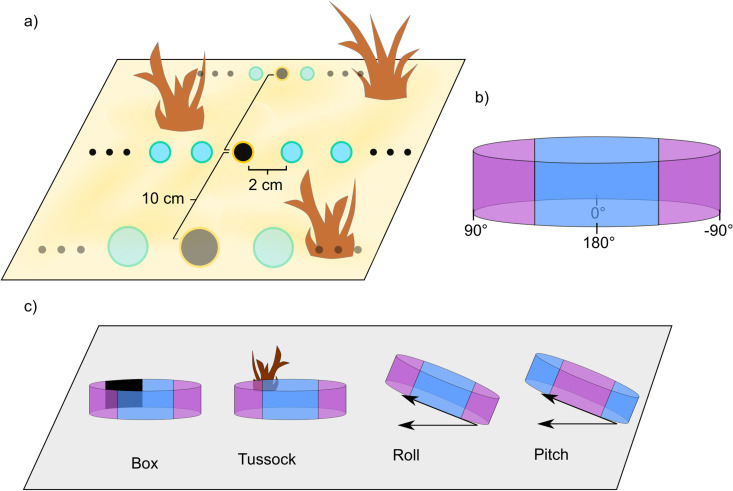
(a) Illustration of the simulated 3D environment in which we took panoramic pictures along a straight route at different snapshot locations (black circles). Each snapshot was then used as a reference image to calculate the rotational image difference function (RIDF) from laterally displaced view locations (turquoise circles). **(b)** Example image from the environment. **(c-d)** Illustration of tilt conditions (roll, pitch) tested in our experiments. The coloured cylinder indicates the wrapped panoramic image. Blue zones indicate areas of the image that shift vertically if pitched. Violet zones indicate areas that shift vertically when rolled. **(e-f)** Illustration of obstruction conditions: a part of the image at a view location was disturbed by a box or tussock.

We will refer to the reference location as the snapshot location and to the image taken at this location as snapshot $X\in {\mathbb{R}}^{M\times N}$. The snapshot is taken facing a given direction which we call the “true orientation” represented by 0°. Similarly, we refer to the displaced location as the view location and to the image seen at this location as view $Y^{\left(d\right)}(r)\in {\mathbb{R}}^{M\times N}$ where *d* is the distance to the snapshot location in cm (see Fig 1(a)) and *r* is the azimuth rotation in degrees. Note that *X* and *Y* may refer to any image representation (e.g., greyscale pixels, wavelet coefficients or binary edge locations).

Using image difference for assessing view similarity we define the rotational image difference function (RIDF) ξ between *X* and *Y*^(*d*)^(*r*) at relative rotation *r* as


$$\xi_{XY^{\left(d\right)}}(r)=\sum_{m,n}^{M,N}|X_{mn}-Y_{mn}^{\left(d\right)}(r)|$$ (1)

where m∈{1,..,M} and n∈{1,..,N} index the image row and column respectively. We denote the rotation where the minimum of the RIDF occurs as r^*^,


$$r^{*}=\underset{r}{argmin}(\xi_{X,Y^{\left(d\right)}}(r))$$ (2)

We then define the absolute angular error for a given displacement *d* as the absolute difference between the true orientation, which by our convention is 0° and r^*^, i.e., $\left|r^{*}\right|$. Small values of $\left|r^{*}\right|$ indicate high performance, as the agent would select a direction of movement that is close to the correct direction. For example $|r^{*}|=10^{\circ }$ corresponds to a 10° error, which would result in 18 cm stray over a travelled distance of 1m. However, note that the general navigation algorithm is iterative and so we would expect the agent to get a new heading at each point. As such whether the error is 9° or 10° is less important than whether there is a failure of navigation, i.e., an erroneous heading overall. Again, if there is a failure, it is not important whether it is 120° or 140° so for this reason we focus on counting the number of errors.

Specifically, if $|r^{*}|>22.5^{\circ }$ we consider the error to be sufficiently large that the location associated with *Y*^(*d*)^ is deemed a “failed location”. The choice of 22.5° gives the agent a 45° sector around the direction parallel to the route which, in observations of robot trials, will often lead to success. Additionally, observing the repeated paths of individual desert ant foragers shows that their variance in directions are in this range [30]. However, as the particular threshold is somewhat arbitrary, we have tested other thresholds and are satisfied that there is no qualitative change to the pattern of results (see section “The effects of resolution on visual navigation”). Note that we count all errors over 22.5° to be failures whether or not the deflection would guide the agent back to the route (i.e., if the test point was to the left of the training route and the error move it rightwards). This is because the ‘correct’ ant-mimicking behaviour is to move parallel to the route [30], and any convergence is accidental. Ants (and robots) do need a mechanism to stop them diverging from routes. Indeed, we are currently investigating separate convergence mechanisms, such as using only the frontal part of the image for matching, a method seen in robotics (e.g., [31,32]). However, our goal in this paper is to test visual compass style route navigation in isolation from visual homing (which does enable convergence as seen in, e.g., [20]).

We calculate the relative number of failed locations *Rel*_*f*_(*d*) which is simply the proportion of failures, for each distance of displacement *d*. Using *Rel*_*f*_(*d*) we characterize the performance of a model as the maximum displacement d^*^ for which *Rel*_*f*_(*d*) < 0.1, which corresponds to the maximum displacement possible such that 10% or less of all positions at that distance failed. Again, the choice of 10% is somewhat arbitrary, but necessary so that we can summarise our results. In this case, 10% was chosen to be a conservative value but, from observation of how *Rel*_*f*_(*d*) increases for increasing *d*, is a point after which *Rel*_*f*_(*d*) for one condition remains consistently above/below other conditions and so does reflect well if one condition is generally better performing than another.

### Visual processing models

As our control condition, we mainly use the “SkyAdjusted” model which uses greyscale images *I* in which sky pixels are set to zero,


$$\begin{array}{c} Z_{m,n}=\left\{ \begin{array}{cc} I_{m,n} & \text{for }I_{m,n}\geq 189\\ 0 & otherwise \end{array}\right. \end{array}$$ (3)

This model adjusts object-to-sky contrast, something known to occur in ants whose UV sensitive vision reliably separates ground from sky. While our decision to set the sky to 0 is somewhat arbitrary and has not been optimised, having sky pixels which are very different to the ground pixels usually performs poorly (e.g., compare SkyAdjusted and GreyScale in section “The effects of resolution on visual navigation”

**Fig 2:**
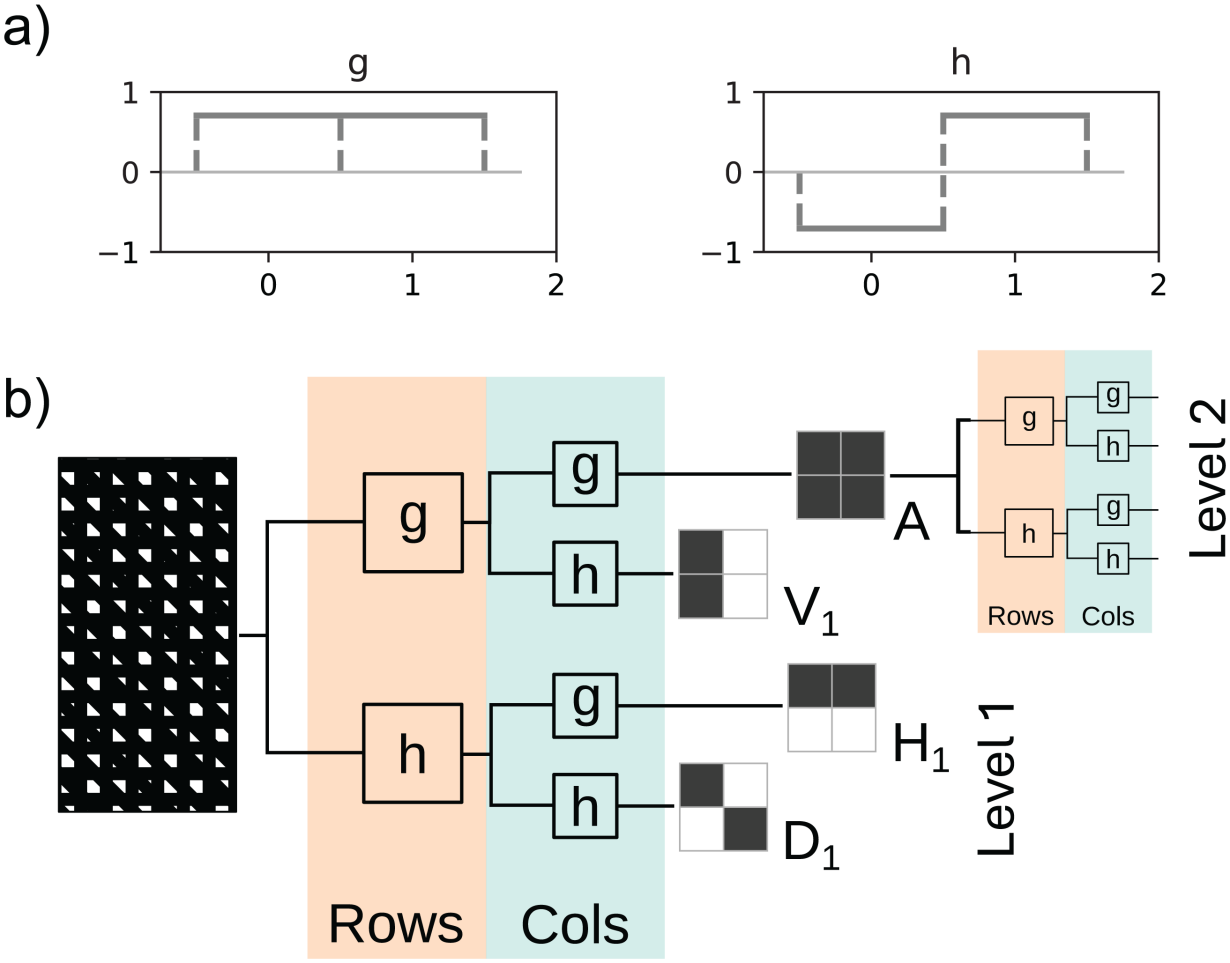
Schematic Illustration of 2-dimensional Separable Discrete Wavelet Transform. **a)** Low pass (*g*) and high pass (*h*) 1D filters of the Haar wavelet (here: y-axis is filter weight, x-axis is pixels). b) separable and iterative 2D discrete wavelet transform. The input signal (an image) is filtered with 1D Haar wavelets (*g* and *h*) applied to rows and to columns sequentially. Depending on the combination of filters *g* and *h*, different 2D filters result (*V*_1_, *H*_1_, *D*_1_ or *A*). The filter *A* results from applying *g* twice and is a simple downsampling of the image, which can be the subject of another wavelet transform, resulting in details of the second level.

For our wavelet models we perform a discrete wavelet transform (DWT) [15]. The DWT utilises a set of two filters: a low pass and a high pass filter that are specific to the mother wavelet that is being used. We chose ‘Haar’ wavelets with *g* = [0.707; 0.707] and *h* = [–0.707; 0.707] as low- and high-pass filter respectively illustrated in Fig 2(a). The low pass filter acts as the scaling function, while the high pass filter acts as the wavelet. The signal is filtered by the high-pass filter to get the “details” and by the low-pass filter to get the “approximation”. The combination of details and approximation form the result of a 1-level wavelet transform. The approximation can now be considered the new signal and the process can be repeated, resulting in details and approximation of a 2-level wavelet transform.

The above procedure describes the DWT of a 1D signal. In this paper the wavelet transform is applied to images which can be considered 2D signals. In a 2D wavelet transform the high and low pass filters are subsequently applied over the rows and columns of the image in varying order to achieve details of different orientation (shown schematically in Fig 2(b)). This process results in four sets of coefficients, three of which contain the orientated details (highest frequencies) in diagonal *D*_*j*_, vertical *V*_*j*_ and horizontal *H*_*j*_ direction of level *j*. For example, by applying the high pass filter to each row of the original image first and then applying the low pass filter to each column of the result, one obtains the high frequencies of the first level *j* = 1 in the rows *V*_1_.

More formally, the wavelet model transforms the original greyscale image *I* by


$$\tilde{Z}=|f_{\circ }(DTW_{L}(I))|$$ (4)

where $\tilde{Z}\in {\mathbb{R}}^{P\times K}$, with *P* = *N*/2^*L*^ and *K* = *M*/2^*L*^ is the wavelet image formed by the magnitudes of coefficients, *DTW*_*L*_(*I*) is the wavelet decomposition applied on *I* up to level *L*, ∘ is a place holder for *V* (vertical), *H* (horizontal) or *D* (diagonal) depending on the orientation of frequency used and *f* is a selector function that selects only the coefficients of the respective level and orientation. For convenience, we will refer to this model (and analogously to other wavelet models) as “V1”. Examples of what the world would look like for V1 can be seen in “Why do wavelets and edges differ?” in Results.

In order to test whether continuous frequency content is advantageous over more binary edge detection, we also compare to the EdgeCanny model which uses the output of Canny edge detection [33] performed on both the snapshot and the view. We used MatLab’s edge function with ’canny’ set to default parameters. We decided to use Canny edge detection over others (e.g., Sobel) because it showed more robust performance in initial testing.

### Visual environment

Our experiments have been conducted in a virtual reconstruction of an ant field site in Spain. The area is flat and characterised by open spaces that contain grass tussocks of varying density [34,35]. Because it is known that the utility of visual information decreases systematically with the distance between a snapshot and a current view [36] we collected 606 (3 straight routes, each with 101 positions) greyscale, 360° panoramic, 0.5°/*px* snapshot images in our environment. For each of these positions, we collected views from laterally displaced locations in 2 cm steps up to 30 cm in both directions. Fig 1(a) shows the world and the organisation of snapshot and sample points. Fig 1(b) shows an example reference image.

We altered the baseline process described above in two principal ways: (1) by bicubic down-sampling to four resolutions (0.5°/*px*, 1°/*px*, 2°/*px* and 4°/*px*) using the MatLab imresize function and (2) by introducing natural perturbations.

The first source of perturbations is the displacement of objects due to, e.g., wind. To account for this, we test two different obstruction conditions (see Fig 1(c), top row). In the “Box” condition, we place a box in the view location occupying approximately 30% of the panoramic image. This approach mimics a broad and general loss of information in the image and is inspired by the experiments described in Wystrach et al. [37]. In the “Tussock” condition, we placed a tussock at a view location in the image where it covers approximately 30% of the panoramic image.

Another imperfection occurring in the real world is the unevenness of the ground leading to tilt which distorts the panoramic image and can affect image matching [38]. Here we separate tilt into pitch, defined as a tilt around an axis orthogonal to the true orientation and roll, defined as tilt around an axis parallel to the true orientation (see Fig 1(c), bottom row). We tilt the agent by 5° and 10° resulting in conditions “Pitch 5°”, “Pitch 10°”, “Roll 5°”, and “Roll 10°”.

**Fig 3:**
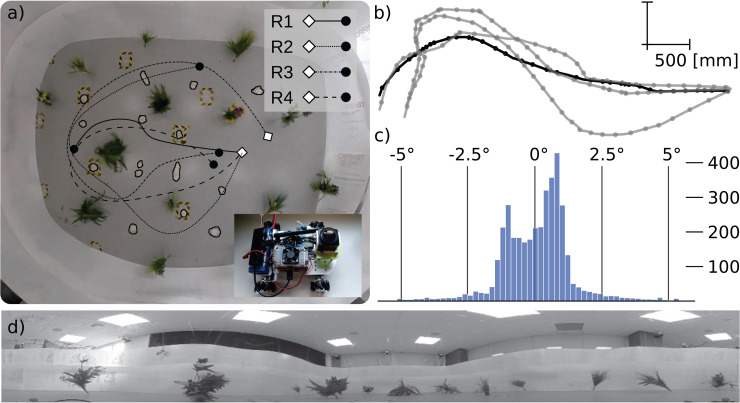
Real world pilot studies. **a)** Top-down view of the arena with schematic depiction of each recorded base route (lines) and hill locations (black outlines). The inset shows the robot used for recording. **b)** Route 1 in its base version (solid black line) and alternative realisations (faded gray lines). Dots show every tenth image-sampling location. **c)** Distribution of measured pitch angles recorded in this environment across all sample locations. **d)** Example image from the environment (the image content above the boundary sheets was cut out and not used for the testing of snapshots and views).

Additionally, to be able to perform more realistic, yet still controllable, experiments, we collected an indoor data set. The data was collected inside a multi-function room inside the University of Sussex. In the room an approximately 4m x 5m rectangular area is surrounded by white plastic sheets, extending up to a height of around 1m. Inside the area, 14 plastic plants of various shapes and similar sizes are placed in a pseudo-random arrangement. Additionally, 15 surface irregularities formed from plasticine were placed in random positions between plants (Fig 3 (a)). In this arena, we recorded five variations of four different routes (an example route with variations is shown in Fig 3(b) using a Vicon 3D Motion Capture System (Vicon Vero, 9 Cameras, Vicon Motion Systems Limited) tracking a mechanum robot with an onboard Kodak Pixpro SP360 panoramic camera, taking 1 picture every 130ms. Each sample point on the base route is considered a training location, while every point on a route version is considered a test location. The plasticine irregularities introduced up to 5° of tilt when passed over (Fig 3 (c)) by the robot. An example view is shown in Fig 3 (d). The degree of tilt of the robot was recorded by an onboard inertial measurement unit (Adafruit 9-DOF Absolute Orientation IMU Fusion Breakout - BNO055).

## Results

To compare between different visual processing methods, we need an objective measure of performance. Any snapshot-based navigational solution relies on the utility of the snapshot, where a high utility snapshot allows deriving the correct action in a larger region around the location it was taken at. This is often referred to as the catchment area (CA) and the size if the CA has thus been the focus of previous work investigating snapshot-based methods. Across all our experiments, we therefore first take an image (snapshot) at a given location with a “true orientation” and a set of images (views) at different distances moving away from the snapshot location perpendicular to the true orientation. We then use the visual compass model to generate an estimated orientation by comparing each view with the snapshot, from which we calculate the absolute angular error between estimate and true orientation. If the absolute angular error is above 22.5° for any view, navigation at this location is considered “failed”.

Our results focus on how the failure rate changes as distance from the snapshot increases for different styles of visual processing and image resolution. We start by analysing the effect of image resolution in isolation, before then examining robustness to tilt and obstacles. Looking purely at which type of processing does well in which condition and for what resolution, summarised in Table 1, it is clear that there is no one “winner” and that the pattern of results is different depending on the different condition. With no perturbations to the images, simple edge detection is best, although the difference in distance at which we get 10% fails for high-resolution wavelets (26.2 vs 25 cm) is not much different. When a tussock is introduced, this pattern is repeated though this time the high-resolution wavelet just wins (16.9 vs 16.3 cm). It also outperforms other methods for all pitch experiments and does creditably in the roll conditions, both of which the simple edge processing cannot cope with. Overall, despite the best wavelet being a lot worse than the best edge-processing (14.9 vs 22.7 cm) in the least-naturalistic Box condition, High-resolution wavelets look to be the best overall, as discussed further below.

**Table 1:** Summarized results. Best performing model per condition-result tuple, in terms of the distance at which 10% of points are failures, highlighted in bold.

Condition \Resolution	0.5°\px	1°\px	2°\px	4°\px
Default	Wavelet 25.01 [cm]	EdgeCanny 24.16 [cm]	**EdgeCanny 26.20 [cm]**	EdgeCanny 23.53 [cm]
Pitch+05	**Wavelet 17.04 [cm]**	SkyAdjusted 15.68 [cm]	SkyAdjusted 14.70 [cm]	SkyAdjusted 13.68 [cm]
Pitch+10	Wavelet 10.24 [cm]	**Wavelet 11.10 [cm]**	Wavelet 9.61 [cm]	Wavelet 8.39 [cm]
Roll+05	Wavelet 19.20 [cm]	**SkyAdjusted 20.10 [cm]**	SkyAdjusted 19.29 [cm]	SkyAdjusted 16.80 [cm]
Roll+10	**SkyAdjusted 14.33 [cm]**	SkyAdjusted 13.88 [cm]	SkyAdjusted 12.36 [cm]	SkyAdjusted 10.10 [cm]
Box	Wavelet 14.92 [cm]	EdgeCanny 18.51 [cm]	**EdgeCanny 22.74 [cm]**	EdgeCanny 17.26 [cm]
Tussock	**Wavelet 16.93 [cm]**	EdgeCanny 15.13 [cm]	EdgeCanny 16.34 [cm]	EdgeCanny 13.96 [cm]

### The effects of resolution on visual navigation

In our first set of results, we compare the performance of our Greyscale, SkyAdjusted, EdgeCanny and Wavelet models as the resolution of the image changes from X to Y degrees. While spatial frequencies in the real world are continuous, perceived images limit the frequency content by their resolution and so frequency-based image encodings will be affected by the resolution of the original image. For a panoramic image, one can obtain the degrees per pixel, which in our experiments are 0.5, 1, 2 and 4 °/*px*.

**Fig 4:**
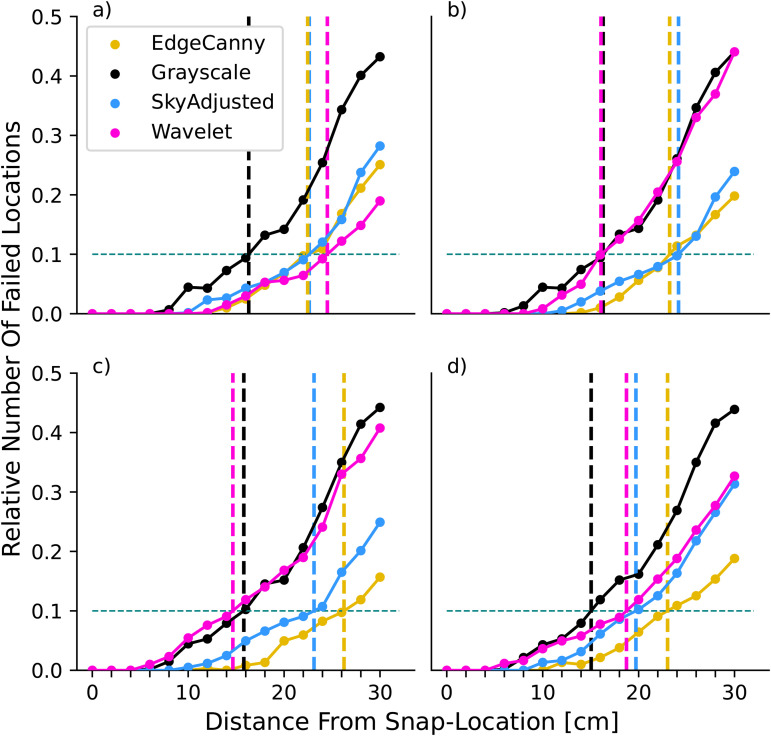
Effect of resolution on model performance. Each panel shows the proportion of failed locations (absolute angular error > 22.5°) as the distance from the snapshot increases for each model (coloured lines, see legend in panel **a)**. Dashed vertical lines indicate the maximum displacement at which 10% of view locations failed (dashed horizontal line) for each model. Results are shown for image resolutions of: **(a)** 0.5°/*px*; **(b)** 1°/*px*, **(c)** 2°/*px*, **(d)** 4°/*px*.

As expected from previous work [36,39], the failure rate for all combinations of model/resolution increases with the distance between current and goal location (Fig 4), but crucially at different rates for the different pre-processing variants. This can be readily appreciated across models and resolutions by comparing the distance at which the failure rate first exceeds 10% (dashed vertical lines in Fig 4, henceforth referred to as *D*_10_).

At the highest resolution, the Wavelet representation performs best, having a lower number of failing locations than other models, especially at larger displacements (Fig 4(a), purple line, *D*_10_ = 25 cm). However, for lower resolutions, the performance of the Wavelet model decreases (Fig 4(b-d)) and it is amongst the worst performing of the models for the lower resolutions. In contrast, EdgeCanny, is not overly affected by resolution. Its peak performance comes at a resolution of 2°/*px* (yellow line, Fig 4(c), *D*_10_ = 26.2 cm), which is the best-performing model overall. Indeed, apart from the highest resolution where Wavelets is slightly better, EdgeCanny outperforms all other models. While the pixel-based representation, SkyAdjusted, comes very close to EdgeCanny at 1° resolution (*D*_10_ = 24.16 vs 24.15 cm, its optimal performance which agrees with [29]), its performance decreases at lower resolutions. Most strikingly, the Greyscale variant, which uses unadjusted sky values, is much worse than the SkyAdjusted variant where sky pixels are 0 (compare black and blue lines in Fig 4) and is pretty much the worst performing model throughout. We, therefore, used SkyAdjusted as our control condition for further tests. Finally, while these results used a failure angle of 22.5°, the same patterns (in terms of the increase in failure rates) are seen when different threshold values are used (see Supporting Information S1 File).

### Why do wavelets and edges differ?

**Fig 5:**
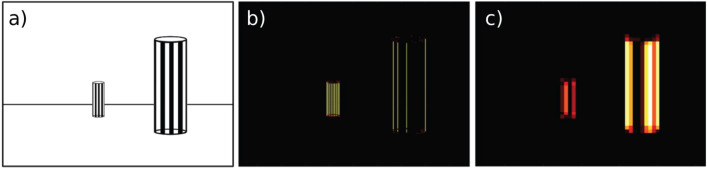
Interaction of spatial frequency and wavelets. **a)** Two pillars with identical spatial structure located at different distances to the observer. **b)** Wavelet representation of vertical features of pillars in a) at high resolution (1320x945px). The distant pillar is represented by more large coefficients (brighter is higher; white max, black 0) than the near pillar. **c)** Wavelet representation of a) after decreasing the resolution by a factor of 4. The near pillar is now represented by larger coefficients (more brighter areas) than the distant one.

Why was the wavelet model the most successful at high resolution but much less so at low resolution, and why is this different to models using simple edges? The answer lies in the fact that, in our environment, there are higher spatial frequencies in the background than in nearby structures because the tussocks that make up the environment have similar spatial structure. This is important, as objects in the background can be more reliable cues for navigation than nearer ones which move more with agent displacement. To illustrate why tussocks at different distances have different wavelet representations depending on resolution, consider two pillars with identical spatial patterns (Fig 5(a)). The more distant pillar has the same spatial frequency as the close pillar, but due to the distance, the perceived frequency is higher (assuming sufficiently high-resolution images that individual edges are resolved), meaning that its wavelet representation has larger coefficients than the one of the nearer pillar (Fig 5(b)). However, at lower resolutions, the spatial structure of the displaced pillar becomes blurry and loses its high-frequency content, so that the nearer pillar is now represented by more large coefficients (Fig 5(c)). As the magnitude and position of the coefficients directly determines the RIDF, at high enough resolution, the wavelet-based visual front end focuses on background structure, while at lower resolution, objects that are close by are most influential. Crucially, this is not the case for an edge-based representation, which has the same number of edges in both cases.

**Fig 6:**
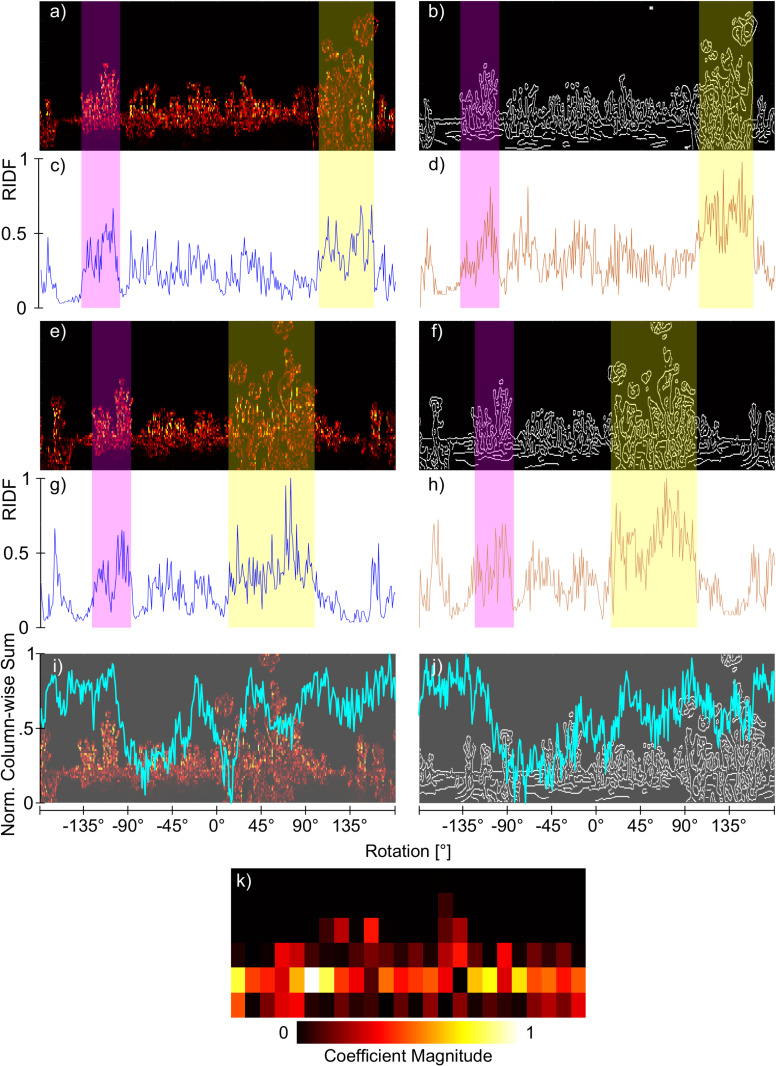
Effects of Wavelet and EdgeCanny visual processing on visual compass robustness. (a, b) Snapshot as represented by wavelets (a) and canny edges (b). (c-d) Normalized column-wise sum of a,b respectively. (e-f) View at nearby location at the same orientation as the snapshot, and hence the correct orientation, when represented by Wavelet (c) and EdgeCanny models. (g-h) Normalized column-wise sum of e,f respectively.**(i-j)** Normalized RIDF (cyan lines) when comparing snapshot with view for Wavelet (i) and EdgeCanny (j) models, superimposed on the view from the nearby location (e-f) but rotated to the best matching orientation as determined by the minimum of the RIDF. In a-h, shaded rectangles highlight the effect of distant (purple) or nearby (yellow) tussocks on the different representations. (k) Magnitude of coefficients for 2-level horizontal details at 4°/*px* resolution.

The differential contribution of distant tussocks to the shape of the RIDF when images are processed into wavelets or edges can be seen in Fig 6 which shows a typical example of a location where the visual compass with wavelet pre-processing recovers bearing correctly, but with the edge model does not. Comparing the Wavelet (Fig 6(a)) and EdgeCanny (Fig 6(b)) representations of the snapshot, we can clearly see that all tussocks contribute but there is a hint that, as in Fig 5, the wavelet representation has a denser concentration of high (yellow) coefficients in the most prominent background tussock (purple shaded rectangle) compared to the foreground tussock (yellow rectangle). This is borne out in Fig 6(c) where the coefficients are summed along each column of the image (an indication of how much different columns contribute to the image representation and subsequent RIDF). Here we see that both distant and near tussocks have similar contributions (compare purple to yellow rectangles in Fig 6(c)), with the denser coefficients of the distant tussock balancing out the nearer tussocks greater size but lower density of coefficients. In contrast, when processed into edges (Fig 6(b)), the size of the near tussock means that it has a greater contribution to the image representation (Fig 6(d)). In the view that is to be matched (Fig 6(e-h)) we see a similar tale: the relative contribution of the foreground versus background tussock to the RIDF is greater in the edge representation than in the wavelets. When the images are compared at different orientations to generate the RIDFs (cyan lines in Fig 6(i-j)), the relatively greater contribution of the background in the wavelet representation means that the best match is near the correct orientation, despite the fact that the nearby tussock has moved a considerable distance (compare the near/far tussock positions in (Fig 6(a,e,i)). In contrast, when matching edges, the near tussock dominates meaning that the best matching orientation from the RIDF is when the image is rotated so that the near tussock matches the snapshot (Fig 6(b,j)) despite the far tussocks being displaced.

**Fig 7:**
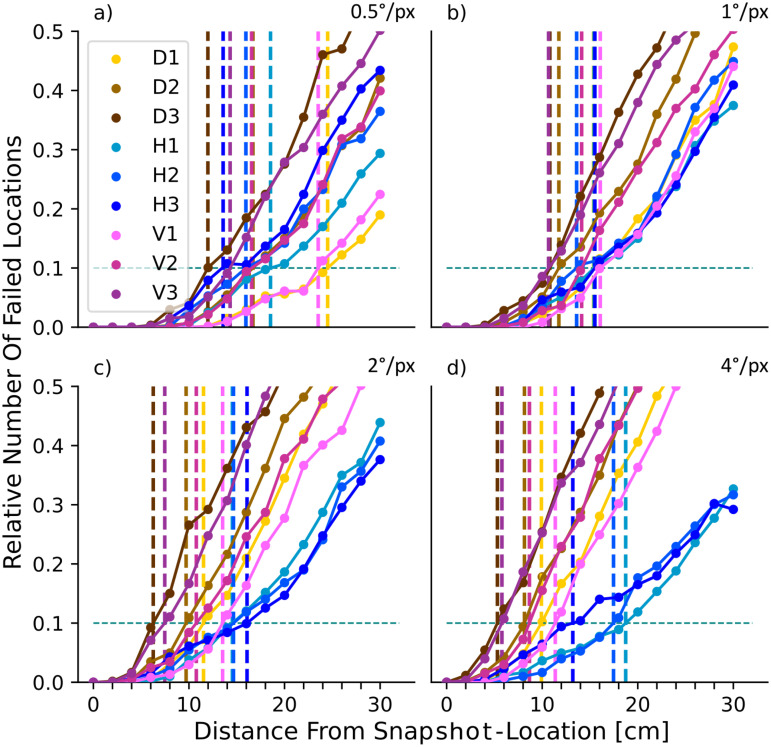
Effect of resolution on separated coefficients of the wavelet analysis. The conventions of the plots follow Fig 4. Vertical wavelets are shown in shades of purple, horizontal wavelets in shades of blue and diagonal wavelets in shades of yellow. Darker shades indicate lower frequency content. (a) Results for 0.5°/*px*. (b) Results for 1°/*px*, (c) Results for 2°/*px*, (d) Results for 4°/*px*). Note how at high resolutions, vertical/diagonal features are most robust against off-route displacement. At lower resolutions, horizontal features are more robust.

In order to investigate whether the distance of objects generating the dominant coefficients is a general driver for bearing recovery performance, we looked at locations where the canny edge model would fail, but the wavelet model would perform well. For this, we restricted our investigation to locations which are at least 20 cm apart to exclude over-representation of local phenomena, and locations where the absolute error of EdgeCanny would surpass 90°. For 22 positions that fit these criteria, we manually determined if close-by or distant tussocks contributed to the shape of the RIDF using the visualisation depicted in Fig 6 (e,f,g,h). At the highest resolution, we were able to identify at least one large tussock formation in the distance which positively affected the alignment with the correct orientation in 16 cases. In the remaining cases, the contribution of distant tussocks could not be unequivocally determined. We repeated this process for lower resolutions looking at 30 positions per resolution. It became evident that here, close-by objects dominated the shape of the RIDF for the wavelet model as well, leading to the observed decrease in performance with decreasing resolution, especially for vertical features (Fig 7(b,c,d)).

Our conclusion that it is the high spatial frequencies that are useful for the Wavelet model is backed up by Fig 7 where we evaluate the contribution of wavelet coefficients of different scales and orientations. At high resolution, we see that the highest frequency vertical and diagonal components are the most effective (Fig 7(a), yellow and pink lines). This makes sense as tussocks grow vertically, so high-frequency content will be represented more by vertical than horizontal coefficients. As the resolution is lowered, the efficacy of vertical and diagonal components decreases while the relative contribution of horizontal components increases (Fig 7, shades of blue). At low resolutions, the horizontal components serve to highlight the overall shape of the skyline (Fig 6(k)), which has been shown to provide navigational information for desert ants [39,40] whose eyes are on the order of 1–4 degrees resolution [28].

Thus, in a route following task based on the visual compass model, high resolution can be used to gain robustness against displacement using naturally occurring, vertically orientated, spatial frequencies of the environment. It is worth noting here that no object detection or distance estimation of landmarks is needed, as these are implicitly defined by their spatial frequency.

### The effect of tilt: Virtual world

In the previous sections, we saw why the use of wavelets as a pre-processing model can improve navigation when compared to no pre-processing and why they produce different results to pre-processing views into edges in a perfectly flat world. As natural environments contain bumps which change an agent’s attitude, and thus views, we repeated our displacement experiments with a modification: at the view location the agent is either tilted upwards (pitch) or sideways (roll) by 5° or 10° as if the agent was on a local-transparent slope.

**Fig 8:**
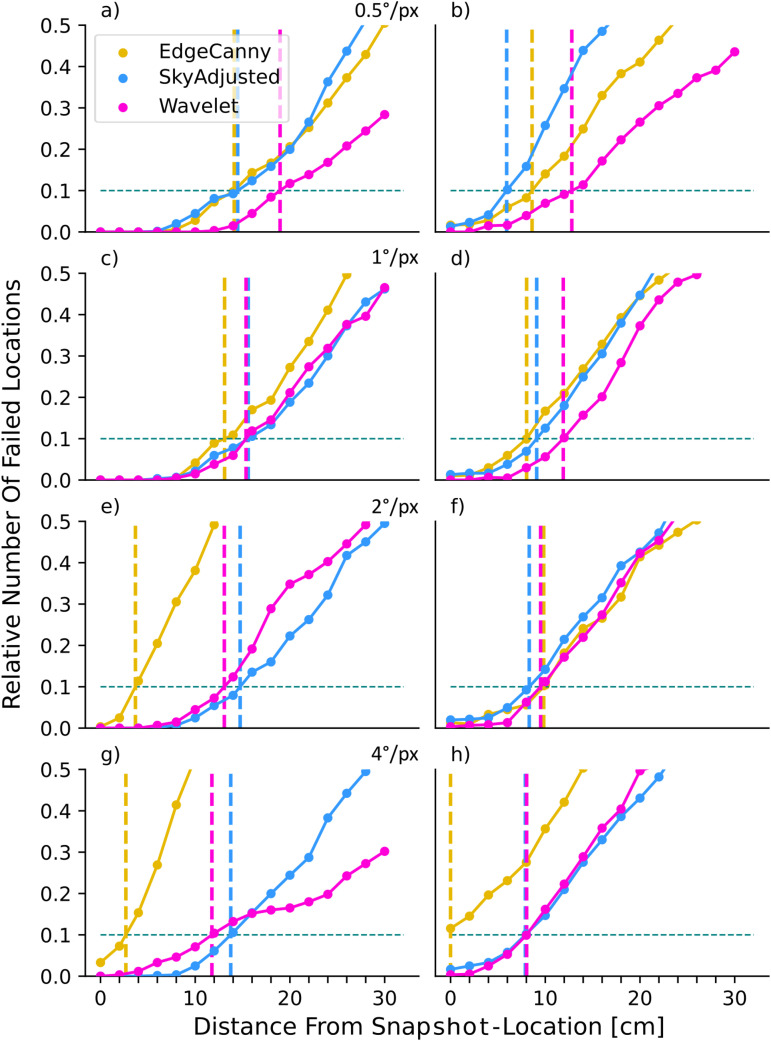
Effect of pitch on model performance at different resolutions. For a description of the plot format see Fig 4. **(a)** 0.5°/*px*, p + 5°. **(b)** 0.5°/*px*, p + 10°. **(c)** 1°/*px*, p + 5°. **(d)** 1°/*px*, p + 10°. **(e)** 2°/*px*, p + 5°. **(f)** 2°/*px*, p + 10°. **(g)** 4°/*px*, p + 5°. **(h)** 4°/*px*, p + 10°.

**Fig 9:**
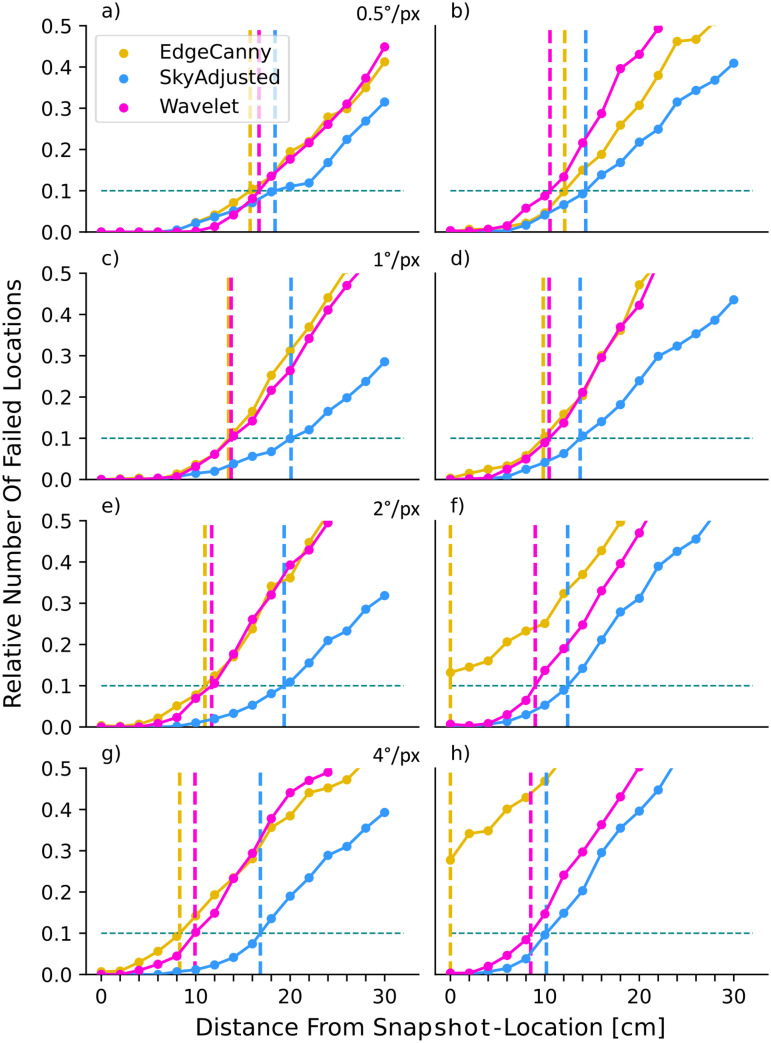
Effect of roll on model performance at different resolutions (for a description of the plot format see Fig 4). **(a)** 0.5°/*px*, r + 5°. **(b)** 0.5°/*px*, r + 10°. **(c)** 1°/*px*, r + 5°. **(d)** 1°/*px*, r + 10°. **(e)** 2°/*px*, r + 5°. **(f)** 2°/*px*, r + 10°. **(g)** 4°/*px*, r + 5°. **(h)** 4°/*px*, r + 10°.

As expected for image matching models, increasing the angle of tilt affects all model performances negatively (Figs 8, 9) but the relative performance of image encodings is different to that seen when views and snapshots were both level. It is notable that for roll and pitch, edge-based pre-processing is the worst-performing model overall (yellow lines in Figs 8, 9).

Considering 5° pitch first (Fig 8, left column), at high resolution the Wavelet model outperforms all other models/resolutions (*D*_10_ = 17 cm). As resolution decreases, however, pixel-based models (SkyAdjusted, blue lines in Fig 8) outperform the others, coming close to the optimal (best *D*_10_ = 15.7 cm) and being relatively insensitive to changing resolution. In contrast, the performance of both edge and wavelet encodings reduces as resolution is lowered, though wavelets are consistently better than edge encodings which show a significant drop in performance for the two lowest resolutions (*D*_10_ of only 3 cm vs 10 for the other models; Fig 8 (e,g)). At 10° pitch we observe, as expected, lower overall performance compared to 5°. As before, the Wavelet models have the best overall performance and are more robust (*D*_10_ from 11.1 to 8.4 cm), performing similarly for the two highest resolutions and outperforming the pixel-based model at all resolutions (Fig 8(b,d)). As resolution decreases, the models perform quite similarly, although again, EdgeCanny performs very poorly at the lowest resolution (Fig 8(f,h))

To understand why these models perform differently it is necessary to consider the effect of pitch on a view. Upward pitch introduces more sky in the “front” and more ground in the “rear” of the perceived image (Fig 1(d)) but the consequences on image difference calculations depend on both location and distance of objects. Distant objects in the frontal visual field tend to be more affected by pitch than near ones because they are generally smaller. However, objects in the lateral visual field are less affected because they are closer to the axis of rotation, and so displacing the agent laterally (as in our tests) results in the lateral parts having similar changes as when there is no pitch. The difference in performance between models is thus a combination of the location of an object in the image (e.g., lateral or frontal) and the distance of this object to the observer. Thus, for the SkyAdjusted model, pitch influences the RIDF strongly only if the original snapshot had large vegetation laterally in front of the agent, as this would lead to aliasing with the increased amount of ground in the rear of the image. The rotational component has little influence, as the relative change in the location of pixels to the side is small, meaning only few pixels are falsely overlapping with sky/tussocks due to image smoothness.

The wavelet model at higher resolutions represents tussocks with coefficients of a magnitude proportional to their distance (spatial frequency) but does under-represent the ground due to lack of texture making it more robust against this form of aliasing. Additionally, distant objects close to the rotational axis of the image distortion remain reliable cues that are represented by large coefficients. Larger, far away tussocks in the tilted-up part of the image are also represented by large coefficients and displaced in image space due to the pitch. However, they tend to not lead to aliasing as they occupy parts of the image that have originally been sky/ground and thus don’t match anything in the original snapshot in the wavelet domain. In order for them to cause aliasing they would need to overlap with a disproportionately large distant tussock, which is unlikely.

EdgeCanny represents ground structure, as well as close and distant objects, equally given the edges generated. At high resolutions, it suffers similar problems as the SkyAdjusted model because it also incorporates the ground structure which can lead to aliasing. At low resolutions, only a few edges make the image, with the main edge being provided by the horizon. Pitch means that the horizon edge overlaps with the few edges provided by tussocks leading to large amounts of aliasing and a decrease in robustness.

Under the influence of roll, the lateral part of the image gets either additional elevation or is lowered, adding to or counteracting the displacement effect. But since the frontal part of the image is close to the axis of rotation, disturbance effects are expected to be less significant. We hence expect the pitch condition to be harder than the roll condition (Fig 1). Indeed, we observe that models in general are more robust against roll than against pitch (see summary in Table 1 and Discussion), as expected. Similar to pitch, we observe model performance decreasing as roll increases (Fig 9, left vs right column). Focusing on 5° roll first, we observe that SkyAdjusted performs more robustly than the frequency models. The SkyAdjusted model performs only slightly worse with 5° roll present than without any tilt (Fig 9(c,e), blue line, $D_{10}\in [10.114.3]$cm). Both frequency models perform similarly and worse than the pixel-based models at all resolutions (Fig 9(a,c,e,g), magenta/yellow lines). At 10° roll performance decreases for all models. Furthermore, it can be seen that models perform more similarly, at least at higher resolutions (Fig 9(b,d)). As resolution decreases, model robustness shows differences (Fig 9(f,h)) with both Wavelet and EdgeCanny performing worse than SkyAdjusted, and EdgeCanny performing notably worse (Fig 9(f,h), yellow line).

In summary, we have observed that the SkyAdjusted model performs consistently over all resolutions and struggles most with pitch. The EdgeCanny model performs very well at all resolutions when no tilt is present, but performance drops notably when tilted, especially at lower resolutions. The Wavelet model works best at high resolutions, where it outperforms the other models consistently. As resolution decreases it tends to be the second-best model, surpassed by either of the other models, depending on the condition.

### The effect of tilt: Real world

**Fig 10:**
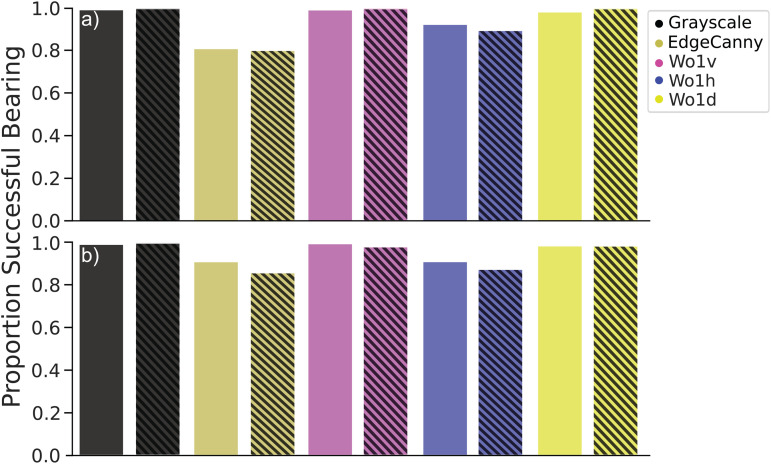
Summary for real world effect of pitch. Locations, where the absolute angular error was below |22.5|° are considered successful. The y-axis shows the proportion of successful locations out of all test locations. Bars show results obtained for different models. Solid bars show results for tilt below |2.5|°. Striped bars show results for tilt above |2.5|°. **(a)** 0.5°/*px*. **(b)** 2°/*px*.

To extend the investigation of tilt further, we performed a similar experiment using our real-world data set. For each test location (i.e., with tilt) we would determine the closest snapshot location and use this snapshot as the reference image for the calculation of the RIDF. Using the absolute angular error we determined the proportion of test locations that resulted in successful bearing recovery (absolute angular error < |22.5°|) over all test locations. We then considered two sets of results, depending on the tilt difference. The first set considered all view locations with a tilt difference between view and snapshot location of <|2.5°| (Fig 10, solid bars). The second set contained results for all test locations where the tilt difference was > |2.5°| (Fig 10, striped bars). Lastly we determined the performance for each model at 0.5°/*px* (Fig 10(a)) and 2°/*px* (Fig 10(b)).

We observe that, in this specific indoor environment, all models tend to perform well. The environment itself contained comparatively few objects (14 tussocks) compared to the virtual world (>100 tussocks). This lack of tussocks results in less visual aliasing, leading to better overall results. However, we do observe differences between the models. Most notably, EdgeCanny (Fig 10, muted yellow) performs noticeably worse than the other models. We also see that at the previously observed sweet spot resolution of 2°/*px* the presence of tilt results in a decrease of performance. We furthermore observe that horizontal wavelets tend to perform worse than other orientations (Fig 10, blue) and show a similar ’dip’ in performance when tilt is present.

### The effect of view obstruction

In the real world, uneven ground is not the only issue that affects a view-matching agent. The surrounding environment might change physically (e.g., objects and vegetation moving or growing, or even being destroyed) and may be illuminated very differently across the day and the seasons, both factors that could lead to inevitable mismatches between a snapshot and a current view. In our desert ant habitat in which objects are tussocks that are similar to one another in shape and size, such issues could pose a severe challenge due to the potential for visual aliasing between tussocks. To investigate how robust the different encodings are to disruptions from objects appearing/disappearing, we repeated the basic displacement experiments but, after displacing the agent, we introduced a new object into the view. While this is a bigger change than objects simply moving, we wanted to push our models as smaller changes would make it difficult to identify differences between them.

In our first condition (box), inspired by ant experiments in which a large rectangular sheet was placed in the world before testing [37], we replaced 25° of the frontal part of the view with black pixels, by setting the pixel values in the relevant image area to zero, as pictured in Fig 1(e). While experimentally motivated, such an obstruction is unlikely to occur in the real world, and so, in the second condition, we introduced a more natural change, specifically, an additional tussock at a distance and orientation such that it covered a similar portion of the visual field as the box (Fig 1(f)). Unlike a box, a tussock would not spontaneously appear in the real world but it might disappear. Because the difference between snapshot and current view is the same as the difference between current view and snapshot, the addition of a tussock in the current view effectively models a tussock disappearing from the snapshot.

**Fig 11:**
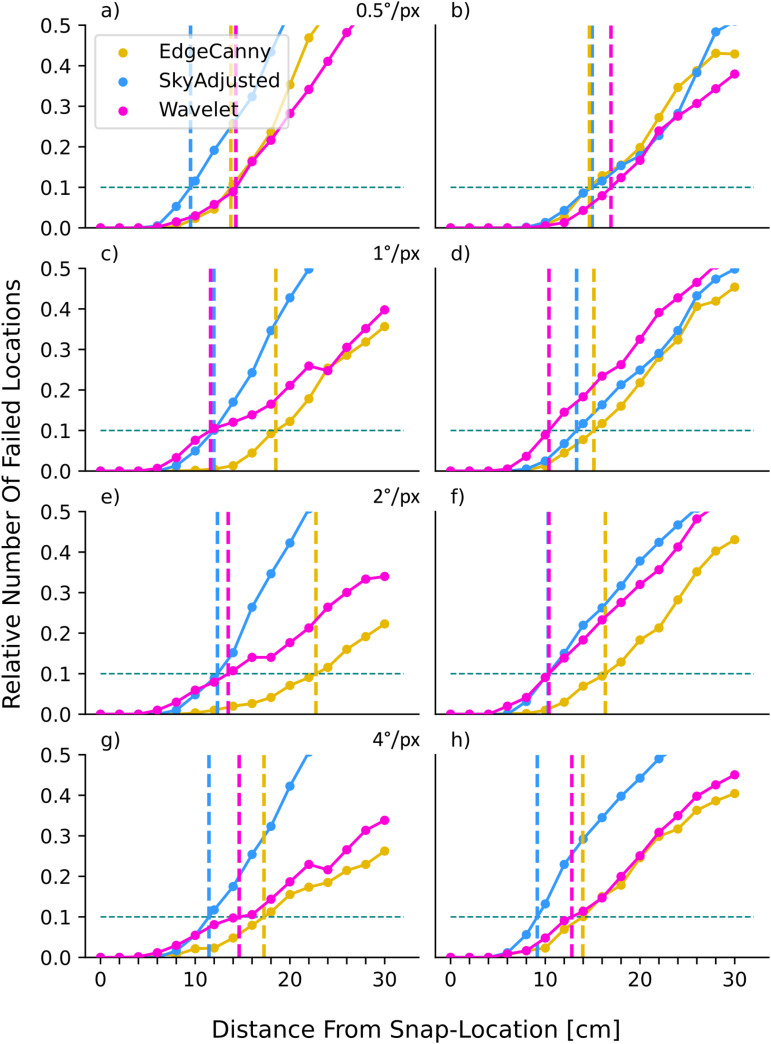
Effect of obstructions on model performance. Each panel shows results for three models (inset key) at different resolutions in scenarios where the view is obtructed by a box (left column) or tussock (right column). **(a)** 0.5°/*px*, Box. **(b)** 0.5°/*px*, Tussock. **(c)** 1°/*px*, Box. **(d)** 1°/*px*, Tussock. **(e)** 2°/*px*, Box. **(f)** 2°/*px*, Tussock. **(g)** 4°/*px*, Box. **(h)** 4°/*px*, Tussock. For description of plot format see Fig 4.

As expected, all models are affected by the introduction of the objects (Fig 11), but there are clear differences between encodings. The pixel-based encoding, SkyAdjusted, is severely impacted, as both objects represent very large visual changes. The box condition is a larger change than the tussock condition as pixels are set to 0. This effect is reflected in our results, as the distance at which the failure rate exceeds 10% reduced in the box condition when compared to the tussock condition at high resolutions (*D*_10_ of 15 vs 9.5 cm; Fig 11, left column, blue line). At lower resolution, the effects of a tussock become more similar to those of the box (as the tussock blurs into a more uniform object), leading to similar results ($D_{10}\approx [10-12]$cm).

For frequency-based models, introducing the box has a different effect than for pixel-based encodings. Adding a box to the view obscures edges behind the box, amounting to removing information from the image in the frequency domain. Adding a tussock both removes information (by obstruction) and adds perturbations (due to introducing new edges). We thus expect the box condition to be less detrimental to the performance of the Wavelet and EdgeCanny models than the tussock condition. While we in general observe less effect of the box on both frequency models than the tussock, (Fig 11, magenta and yellow lines) the EdgeCanny model is less affected by obstruction-perturbations than the Wavelet model. Indeed, EdgeCanny outperforms all models, sometimes by a lot (peak *D*_10_ = 22.7 vs a best of 14.3 cm across the others), at all but the highest resolution suggesting that ignoring a few edges at lower resolutions does not impact performance strongly. In contrast, for the wavelet model, as resolution decreases, the ill-placed tussock evokes large coefficients, leading to aliasing issues (see Fig 5 for explanation) until the lowest resolution where most edges are formed by the skyline. However at high resolutions the wavelet is slightly better than edges (*D*_10_ = 16.9 vs 16.3 cm).

## Discussion

**Fig 12:**
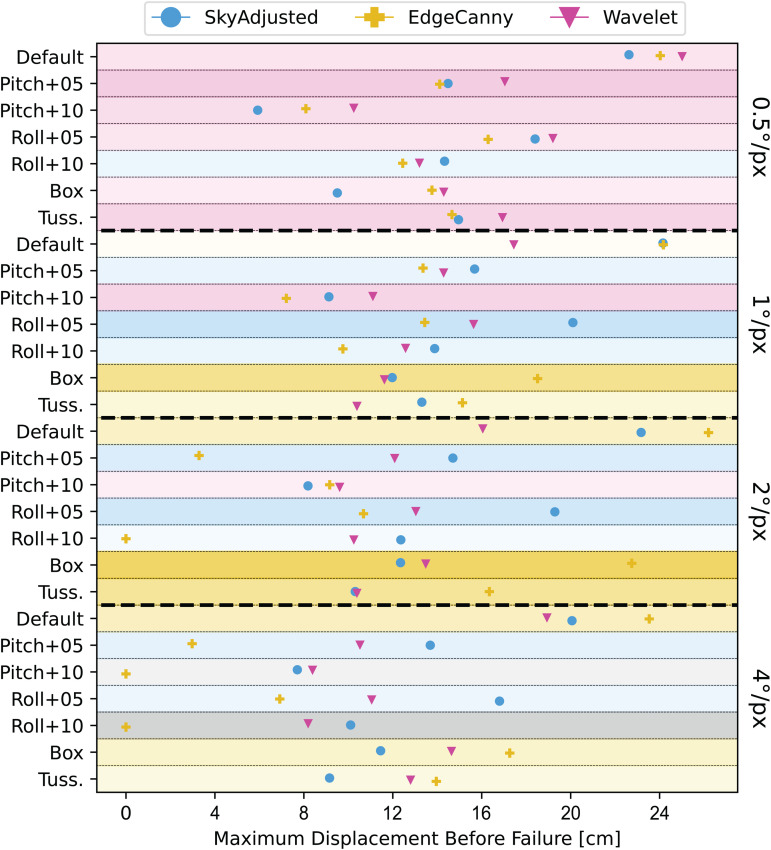
Results summary. Depicted is the displacement before failure (> 10% of all tested locations failed) for each condition (rows) and model (markers). The further to the right a marker, the better the model performed. Each row is highlighted by the colour corresponding to the best-performing model. The intensity of the highlight is scaled by the difference between the best-performing and second-best-performing model (dark colour indicates the model is outperforming others by a large margin).

In this paper we have investigated the efficacy of localized frequency content for visual bearing recovery, comparing two frequency-based models (EdgeCanny, Wavelet) and one pixel-based model (SkyAdjusted) in a virtual environment under different conditions designed to mimic those under which image-matching algorithms might fail. The results are summarised in Fig 12. Based on our preliminary work [27], we expected that the Wavelet model would outperform pixel-based models and this was indeed the case at the highest resolution. However, there was an unexpected interaction between visual resolution, the visual processing algorithm used, and the nature of the challenge faced by the algorithm. For the wavelet model, at high resolution, vertical wavelet features are particularly robust against displacement, tilt, and obstructions, outperforming EdgeCanny and SkyAdjusted models (Fig 12, top), but at lower resolutions, performance decreases and we observed that horizontal features are more reliable than vertical ones (Fig 7). In contrast, in both the default condition and with obstructions, the EdgeCanny model performs best at low resolution (2°/*px*) and is the best-performing model overall. However, it severely struggles in the presence of tilt with by far the worst performance. Finally, our non-frequency-based SkyAdjusted model is least affected by changes in resolution, performing best at 2°/*px* and dealing very well with tilt but is outperformed by both frequency models when it comes to obstructions. Overall, taking account of all conditions, we feel that wavelet-based encoding is a good compromise in terms of performance and robustness to real-world noise and is a better choice than pixel-based encoding. However, simpler frequency-based encodings such as EdgeCanny can outperform wavelets in some scenarios and so this work mainly highlights that frequency encoding in general is important for bearing recovery.

Our work adds to the body of work investigating frequency-based encodings for view-based homing and navigation by looking in detail at different frequency models and how they react to natural objects and disturbances. [26] showed that the Fourier coefficients of an image can be used to home to a single discrete snapshot location in a goal-searching task. Stone et al. [21] as well as Sun et al. [22] have shown that this also holds true for spherical images if Zernike moments are selected as the image basis. While the aforementioned work limited itself to rotational invariant frequency decomposition, meaning that the location of a given frequency in image space cannot be determined, Lee et al. [23] showed that localised frequency decomposition in the form of random Haar-like wavelet filters can be used to represent images such that successful homing is possible. By further investigating the role of frequency information from higher resolutions than ∼1px/° and using localized and orientated frequency components, we have been able to determine that at high resolutions, high vertical frequencies are sufficient to recover visual bearing. In an environment which is dominated by very similar structures (e.g., tussocks), objects in the background lead to higher coefficients, strongly influencing the RIDF and the bearing recovered, yet these distant objects are less influenced by displacement and so more reliable as the agent moves. This leads to a natural focus on background features at high resolutions. These objects tend to be found close to the horizon line, mostly within the same region of interest selected by [26] for their experiments and also the region of the world for which ant eyes have the highest resolution [41].

We also showed that the orientation, scale and localisation of frequency content in an image can be crucial for recovering a correct bearing in the face of perturbations due to uneven ground. A physical body can, however, provide more than just a vessel for sensory and computational modules: it can mitigate problems visual processing might encounter. For example, sophisticated stabilization methods can negate the effects of tilt on a robot [42] and ants are capable of some degree of head stabilization as well [43] although this is not always evident [38]. One can also take into account behavioural routines a physical body could perform in order to increase the visual information gained. Indeed, any form of locomotion could be adapted to deal with unfortunate view locations by simply re-positioning the agent slightly.

When considering how these findings should be applied to the design of robotic navigating agents, it is interesting to think about what choices one would make for resolution and visual processing algorithms. A robotic agent for example can potentially have access to any resolution we investigated, as well as every visual processing model. However, equipping an agent with an extensive number of cameras or running multiple processes on images drains energy and reduces operation time. Some robots need “high” resolution cameras to perform additional tasks such as pattern recognition [44] or anomaly detection in images [45]. When unifying these tasks through one camera, our results suggest that a 1-level vertical wavelet-based visual processing model would provide the best route following performance (see Fig 12(a), magenta), as it performs consistently across tilt and obstruction perturbations. When designing a robot that uses visual input only for route following, picking a lower resolution (here 1°/*px* or 2°/*px*) camera is more attractive. In this case, knowledge about the environment can be used to determine what visual processing to use. Our findings indicate that in an environment that is unlikely to tilt the agent (such as a street) but might include movable obstructions (such as parked cars) the use of canny edge detection can lead to more robust route following performance (see Fig 12(b-c), yellow). If, on the other hand, the environment is likely to introduce tilt on the agent but is unlikely to have a high amount of obstruction (e.g., a forest glade) our results recommend using a sky-contrast-based approach (SkyAdjusted) for the best route following performance (see Fig 12(b-c), blue).

Visual sensors with resolutions of 4°/*px* or lower are not often relevant for robotics, but resemble the visual apparatus of desert ants [28]. Indeed, in accordance with previous modelling results [29], the pixel-based model performed best at a resolution of 2°/*px* which is around the resolution of ant eyes when viewing the horizon. Further, structural analysis of the visual system of insects in general provides evidence for multi-scale representations in the visual system [12,46–49], which could contain frequency-encoded versions of the image. Such a multi-scale image representation is similar to the wavelet approach we investigated here, allowing us to understand how multi-scale image representations are beneficial. We also highlight that in the condition where part of the view is occluded, the performance of frequency-based models was not affected as much as the pixel-based model. The Box condition for the occlusion trials was inspired by experiments with ants when parts of the ants’ surrounding environment were blocked with a large dark cloth shield [37]. They observed that ants increased their scanning behaviour (indicating uncertainty) with the shield present but were otherwise less disturbed than one might expect from such a large change. Our results suggest that using a frequency-based representation reduces adverse impacts when such a significant disruption is in place.

The aim of this paper was to elucidate the strengths and weaknesses of different visual processing approaches for route following, especially focusing on multi-scale frequency analysis. We found that the value of orientated, localized frequency information depends on the visual apparatus as well as the environment. We have been able to identify and describe how exactly these frequencies influence the RIDF and as such the bearing recovery performance for different resolutions. As such we provide a thorough analysis of the role of frequency in deriving bearing information from a visual compass in the face of common real-world perturbations. For robots, our work suggests that these perturbations can be at least partly overcome by selecting a beneficial image representation. For ants, our results prompt us to investigate whether aspects of insects’ visual systems could serve as implementations of a frequency-based encoding, thus boosting the performance of simple yet effective navigational algorithms.

A natural extension of this work will include investigations in a real-world environment. This can be done on a real-world data set or with an embedded system such as a robot that needs to recover its bearing. A route-following agent is facing additional challenges not investigated here. For example, in this work, the reference snapshot was always assumed to be known. However, a real agent would need to select a snapshot from a set of snapshots stored in memory. Selecting the wrong snapshot can be seen as memory aliasing and would result in the calculation of a meaningless direction. Hence, an extension of the experiments presented here with a focus on the interaction between memory aliasing and frequency representation would elevate our understanding towards a competent holistic route following algorithm.

## Supporting information

S1 File Effect of changing the angular error threshold on number of failed locations. (DOCX)

## References

[1] Hölldobler B, Wilson EO, et al. The ants. Harvard University Press; 1990.

[2] Kohler M, Wehner R. Idiosyncratic route-based memories in desert ants, Melophorus bagoti: how do they interact with path-integration vectors?. Neurobiol Learn Memor. 2005;83(1):1–12. 10.1016/j.nlm.2004.05.011 15607683

[3] Collett T, Graham P, Heinze S. The neuroethology of ant navigation. Curr Biol. 2025;35(3):R110–24. doi: 10.1016/j.cub.2024.12.034 39904309

[4] Zhang AM, Kleeman L. Robust Appearance Based Visual Route Following for Navigation in Large-scale Outdoor Environments. Int J Robot Res. 2009;28(3):331–56. doi: 10.1177/0278364908098412

[5] Baddeley B, Graham P, Husbands P, Philippides A. A model of ant route navigation driven by scene familiarity. PLoS Comput Biol. 2012;8(1):e1002336. doi: 10.1371/journal.pcbi.1002336 22241975 PMC3252273

[6] Ardin P, Peng F, Mangan M, Lagogiannis K, Webb B. Using an Insect Mushroom Body Circuit to Encode Route Memory in Complex Natural Environments. PLoS Comput Biol. 2016;12(2):e1004683. doi: 10.1371/journal.pcbi.1004683 26866692 PMC4750948

[7] Amin AA, Kagioulis E, Domcsek ADN, Nowotny T, Graham P, Philippides A. Robustness of the Infomax Network for View Based Navigation of Long Routes. In: ALIFE 2023: Ghost in the Machine: Proceedings of the 2023 Artificial Life Conference. MIT Press; 2023.

[8] Jesusanmi OO, Amin AA, Domcsek N, Knight JC, Philippides A, Nowotny T, et al. Investigating visual navigation using spiking neural network models of the insect mushroom bodies. Front Physiol. 2024;15:1379977. doi: 10.3389/fphys.2024.1379977 38841209 PMC11151298

[9] Gattaux GG, Wystrach A, Serres JR, Ruffier F. Route-centric ant-inspired memories enable panoramic route-following in a car-like robot. Nat Commun. 2025;16(1):8328. doi: 10.1038/s41467-025-62327-3 40993110 PMC12460611

[10] Amin AA, Philippides A, Graham P. Ant visual route navigation: How the fine details of behaviour promote successful route performance and convergence. PLoS Comput Biol. 2025;21(9):e1012798. doi: 10.1371/journal.pcbi.1012798 40929289 PMC12445746

[11] Seelig JD, Jayaraman V. Feature detection and orientation tuning in the Drosophila central complex. Nature. 2013;503(7475):262–6. doi: 10.1038/nature12601 24107996 PMC3830704

[12] Roper M, Fernando C, Chittka L. Insect Bio-inspired Neural Network Provides New Evidence on How Simple Feature Detectors Can Enable Complex Visual Generalization and Stimulus Location Invariance in the Miniature Brain of Honeybees. PLoS Comput Biol. 2017;13(2):e1005333. doi: 10.1371/journal.pcbi.1005333 28158189 PMC5291356

[13] Cooley JW, Tukey JW. An algorithm for the machine calculation of complex Fourier series. Math Comp. 1965;19(90):297–301. doi: 10.1090/s0025-5718-1965-0178586-1

[14] Khotanzad A, Hong YH. Invariant image recognition by Zernike moments. IEEE Trans Pattern Anal Machine Intell. 1990;12(5):489–97. doi: 10.1109/34.55109

[15] Mallat SG. Multifrequency channel decompositions of images and wavelet models. IEEE Trans Acoust Speech Signal Process. 1989;37(12):2091–110. doi: 10.1109/29.45554

[16] Haar A. Zur Theorie der orthogonalen Funktionensysteme. Göttingen: Georg-August-Universität; 1909.

[17] Menegatti E, Maeda T, Ishiguro H. Image-based memory for robot navigation using properties of omnidirectional images. Robot Autonom Syst. 2004;47(4):251–67. doi: 10.1016/j.robot.2004.03.014

[18] Stürzl W, Mallot HA. Efficient visual homing based on Fourier transformed panoramic images. Robot Autonom Syst. 2006;54(4):300–13. doi: 10.1016/j.robot.2005.12.001

[19] Stone T, Differt D, Milford M, Webb B. Skyline-based localisation for aggressively manoeuvring robots using UV sensors and spherical harmonics. In: 2016 IEEE International Conference on Robotics and Automation (ICRA), 2016. 5615–22. 10.1109/icra.2016.7487780

[20] van Dijk T, De Wagter C, de Croon GC. Visual route following for tiny autonomous robots. Sci Robot. 2024;9(92):eadk0310. doi: 10.1126/scirobotics.adk0310 39018372

[21] Stone T, Mangan M, Wystrach A, Webb B. Rotation invariant visual processing for spatial memory in insects. Interface Focus. 2018;8(4):20180010. doi: 10.1098/rsfs.2018.0010 29951190 PMC6015815

[22] Sun X, Yue S, Mangan M. A decentralised neural model explaining optimal integration of navigational strategies in insects. Elife. 2020;9:e54026. doi: 10.7554/eLife.54026 32589143 PMC7365663

[23] Lee C, Kim D. Visual Homing Navigation With Haar-Like Features in the Snapshot. IEEE Access. 2018;6:33666–81. doi: 10.1109/access.2018.2842679

[24] Stürzl W, Zeil J. Depth, contrast and view-based homing in outdoor scenes. Biol Cybern. 2007;96(5):519–31. doi: 10.1007/s00422-007-0147-3 17443340

[25] Kagioulis E, Philippides A, Graham P, Knight JC, Nowotny T. Insect inspired view based navigation exploiting temporal information. In: Conference on Biomimetic and Biohybrid Systems. Springer; 2020. p. 204–16.

[26] Stürzl W, Mallot HA. Efficient visual homing based on Fourier transformed panoramic images. Robot Autonom Syst. 2006;54(4):300–13. doi: 10.1016/j.robot.2005.12.001

[27] Meyer S, Nowotny T, Graham P, Dewar A, Philippides A. Snapshot Navigation in the Wavelet Domain. Conference on Biomimetic and Biohybrid Systems. Springer International Publishing; 2020. p. 245–56. 10.1007/978-3-030-64313-3_24

[28] Schwarz S, Narendra A, Zeil J. The properties of the visual system in the Australian desert ant Melophorus bagoti. Arthropod Struct Dev. 2011;40(2):128–34. doi: 10.1016/j.asd.2010.10.003 21044895

[29] Wystrach A, Dewar A, Philippides A, Graham P. How do field of view and resolution affect the information content of panoramic scenes for visual navigation? A computational investigation. J Comp Physiol A Neuroethol Sens Neural Behav Physiol. 2016;202(2):87–95. doi: 10.1007/s00359-015-1052-1 26582183 PMC4722065

[30] Mangan M, Webb B. Spontaneous formation of multiple routes in individual desert ants (Cataglyphis velox). Behav Ecol. 2012;23(5):944–54. doi: 10.1093/beheco/ars051

[31] Krajnik T, Majer F, Halodova L, Vintr T. Navigation without localisation: reliable teach and repeat based on the convergence theorem. In: 2018 IEEE/RSJ International Conference on Intelligent Robots and Systems (IROS), 2018. p. 1657–64. 10.1109/iros.2018.8593803

[32] Dall’Osto D, Fischer T, Milford M. Fast and robust bio-inspired teach and repeat navigation. In: 2021 IEEE/RSJ International Conference on Intelligent Robots and Systems (IROS). IEEE; 2021. p. 500–7.

[33] Canny J. A computational approach to edge detection. IEEE Trans Pattern Anal Mach Intell. 1986;8(6):679–98. doi: 10.1109/tpami.1986.4767851 21869365

[34] Risse B, Mangan M, Stürzl W, Webb B. Software to convert terrestrial LiDAR scans of natural environments into photorealistic meshes. Environ Modell Softw. 2018;99:88–100. doi: 10.1016/j.envsoft.2017.09.018

[35] Stankiewicz J, Webb B. Looking down: a model for visual route following in flying insects. Bioinspir Biomim. 2021;16(5):055007. doi: 10.1088/1748-3190/ac1307 34243169

[36] Zeil J, Hofmann MI, Chahl JS. Catchment areas of panoramic snapshots in outdoor scenes. J Opt Soc Am A Opt Image Sci Vis. 2003;20(3):450–69. doi: 10.1364/josaa.20.000450 12630831

[37] Wystrach A, Philippides A, Aurejac A, Cheng K, Graham P. Visual scanning behaviours and their role in the navigation of the Australian desert ant Melophorus bagoti. J Comp Physiol A Neuroethol Sens Neural Behav Physiol. 2014;200(7):615–26. doi: 10.1007/s00359-014-0900-8 24682419

[38] Ardin P, Mangan M, Wystrach A, Webb B. How variation in head pitch could affect image matching algorithms for ant navigation. J Comp Physiol A Neuroethol Sens Neural Behav Physiol. 2015;201(6):585–97. doi: 10.1007/s00359-015-1005-8 25895895 PMC4439443

[39] Philippides A, Baddeley B, Cheng K, Graham P. How might ants use panoramic views for route navigation? J Exp Biol. 2011;214(Pt 3):445–51. doi: 10.1242/jeb.046755 21228203

[40] Graham P, Cheng K. Ants use the panoramic skyline as a visual cue during navigation. Curr Biol. 2009;19(20):R935-7. doi: 10.1016/j.cub.2009.08.015 19889365

[41] Zollikofer C, Wehner R, Fukushi T. Optical scaling in conspecific Cataglyphis ants. J Exp Biol. 1995;198(Pt 8):1637–46. doi: 10.1242/jeb.198.8.1637 9319542

[42] Bereska D, Daniec K, Fraś S, Jedrasiak K, Malinowski M, Nawrat A. System for multi-axial mechanical stabilization of digital camera. In: Vision Based Systemsfor UAV Applications. Springer; 2013. p. 177–89.

[43] Raderschall CA, Narendra A, Zeil J. Head roll stabilisation in the nocturnal bull ant Myrmecia pyriformis: implications for visual navigation. J Exp Biol. 2016;219(Pt 10):1449–57. doi: 10.1242/jeb.134049 26994172

[44] de la Escalera A, Armingol JM, Mata M. Traffic sign recognition and analysis for intelligent vehicles. Image Vis Comput. 2003;21(3):247–58. doi: 10.1016/s0262-8856(02)00156-7

[45] Nakahata MT, Thomaz LA, da Silva AF, da Silva EAB, Netto SL. Anomaly detection with a moving camera using spatio-temporal codebooks. Multidim Syst Sign Process. 2017;29(3):1025–54. doi: 10.1007/s11045-017-0486-8

[46] Seelig JD, Jayaraman V. Neural dynamics for landmark orientation and angular path integration. Nature. 2015;521(7551):186–91. doi: 10.1038/nature14446 25971509 PMC4704792

[47] Strother JA, Nern A, Reiser MB. Direct observation of ON and OFF pathways in the Drosophila visual system. Curr Biol. 2014;24(9):976–83. doi: 10.1016/j.cub.2014.03.017 24704075

[48] Paulk AC, Gronenberg W. Higher order visual input to the mushroom bodies in the bee, Bombus impatiens. Arthropod Struct Dev. 2008;37(6):443–58. doi: 10.1016/j.asd.2008.03.002 18635397 PMC2571118

[49] Dewar ADM, Wystrach A, Philippides A, Graham P. Neural coding in the visual system of Drosophila melanogaster: How do small neural populations support visually guided behaviours?. PLoS Comput Biol. 2017;13(10):e1005735. doi: 10.1371/journal.pcbi.1005735 29016606 PMC5654266

