# Supplementary material for: Wavelet-based visual compass
Source: PLoS One. 2026 Apr 7;21(4):e0344575. doi: 10.1371/journal.pone.0344575 (PMC13056182; doi:10.1371/journal.pone.0344575)
Supplement: S1 File — (DOCX) [file pone.0344575.s001.docx]

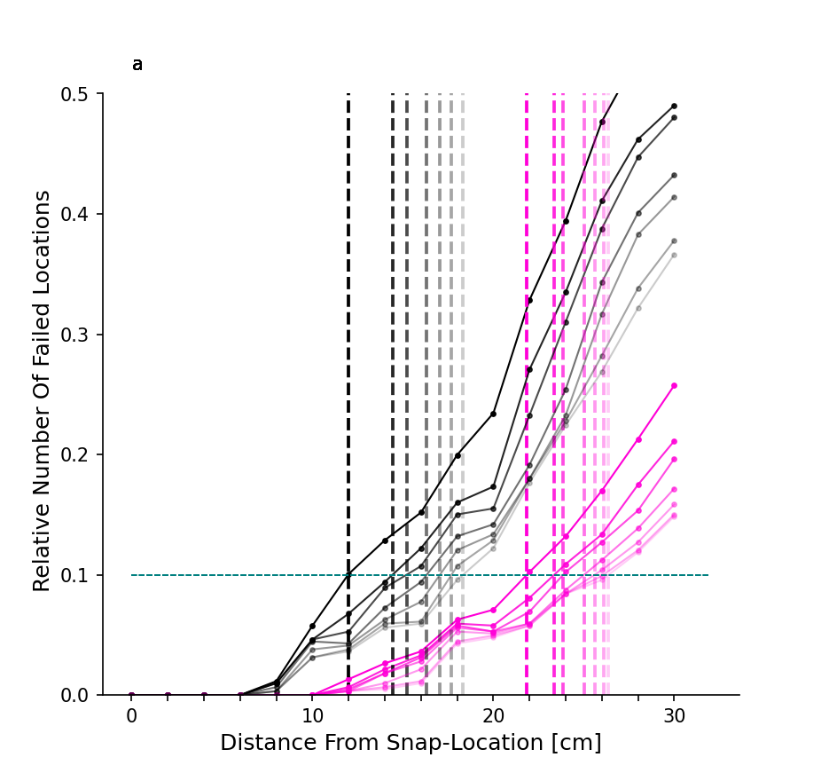


Supplemental Figure 1: Effect of the angular error threshold on the number of failed locations for the wavelet model (magenta lines) and grey-scale model (black lines) for images at a resolution of 0.5*°*/pixel. Angular thresholds are (from top to bottom of each set of lines): *15°; 17.5°; 20°; 22.5°; 25°; 27.5°; 30°.* While the position of the lines change, they do so quite regularly and so, compared to Fig 4, there would be no qualitative change in results. This can be seen in more detail in the Supplemental gif below


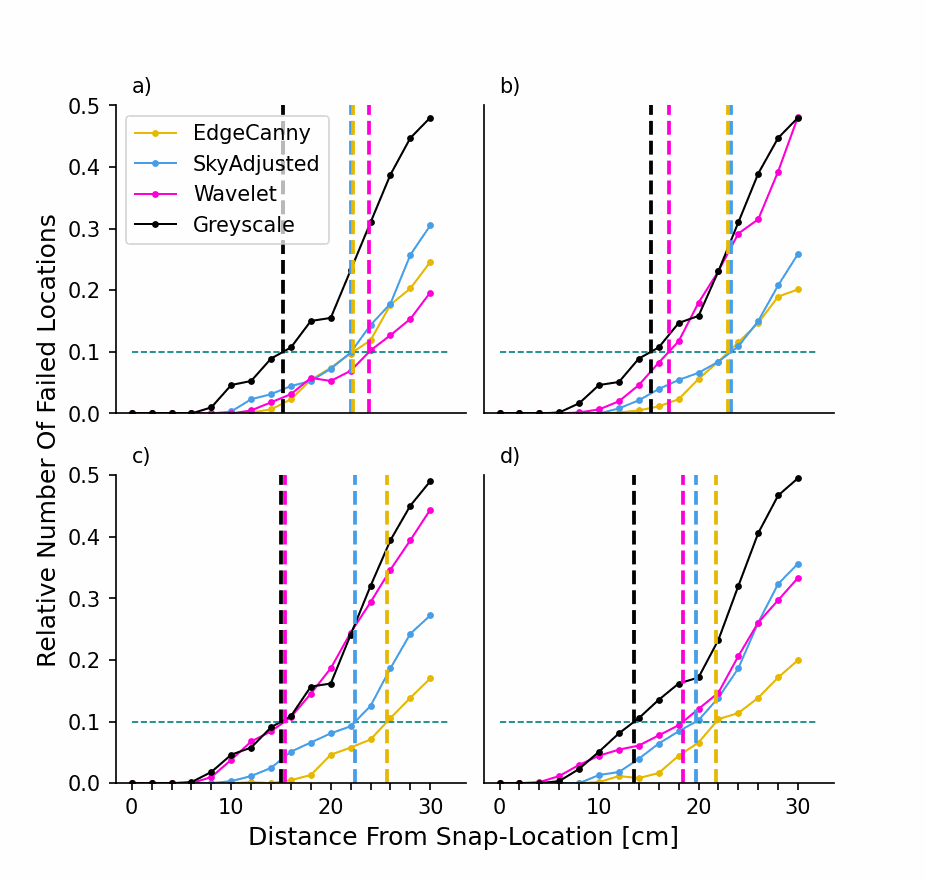


Supplemental gif: This shows the effect on all the results of Fig 4 of changing the angular threshold for failure from ): *15°; 17.5°; 20°; 22.5°; 25°; 27.5°; 30°*  and clearly shows that the overall pattern of results remains unchanged
